# Invasive Assessment of Coronary Microcirculation: A State-of-the-Art Review

**DOI:** 10.3390/diagnostics14010086

**Published:** 2023-12-30

**Authors:** Luca Ciaramella, Luigi Di Serafino, Lucia Mitrano, Maria Luisa De Rosa, Carlo Carbone, Francesco Saverio Rea, Salvatore Monaco, Maria Scalamogna, Plinio Cirillo, Giovanni Esposito

**Affiliations:** Department of Advanced Biomedical Sciences, University of Naples Federico II, Via Pansini 5, 80131 Naples, Italy; luca.ciaramella@unina.it (L.C.); lucia.mitrano@unina.it (L.M.); marialuisa.derosa@unina.it (M.L.D.R.); carlo.carbone@unina.it (C.C.); francesco.rea@unina.it (F.S.R.); salvatore.monaco@unina.it (S.M.); maria.scalamogna@libero.it (M.S.); plinio.cirillo@unina.it (P.C.); espogiov@unina.it (G.E.)

**Keywords:** coronary flow reserve, fractional flow reserve, index of microcirculatory resistance, resistive reserve ratio, absolute coronary flow, microcirculatory reserve ratio

## Abstract

A significant proportion of patients presenting with signs and symptoms of myocardial ischemia have no “significant” epicardial disease; thereby, the assessment of coronary microcirculation gained an important role in improving diagnosis and guiding therapy. In fact, coronary microvascular dysfunction (CMD) could be found in a large proportion of these patients, supporting both symptoms and signs of myocardial ischemia. However, CMD represents a diagnostic challenge for two main reasons: (1) the small dimension of the coronary microvasculature prevents direct angiographic visualization, and (2) despite the availability of specific diagnostic tools, they remain invasive and underused in the current clinical practice. For these reasons, CMD remains underdiagnosed, and most of the patients remain with no specific treatment and quality-of-life-limiting symptoms. Of note, recent evidence suggests that a “full physiology” approach for the assessment of the whole coronary vasculature may offer a significant benefit in terms of symptom improvement among patients presenting with ischemia and non-obstructive coronary artery disease. We analyze the pathophysiology of coronary microvascular dysfunction, providing the readers with a guide for the invasive assessment of coronary microcirculation, together with the available evidence supporting its use in clinical practice.

## 1. Introduction

A large proportion of patients presenting with typical chest pain and signs of myocardial ischemia is found with non-obstructing epicardial disease, but coronary microvascular dysfunction (CMD) may be present. However, CMD represents a diagnostic challenge; therefore, the purpose of this review is to describe the pathophysiological mechanisms of CMD and the available invasive diagnostic modalities.

Two different invasive tools are currently available for the assessment of coronary microcirculation: Doppler-based and thermodilution-derived tools. For each of them, we will discuss the measurement principles, advantages, and limitations. Finally, we will provide the readers with an overview of the currently available evidence for their use in clinical practice.

## 2. The Coronary Circulation

**Figure 1:**
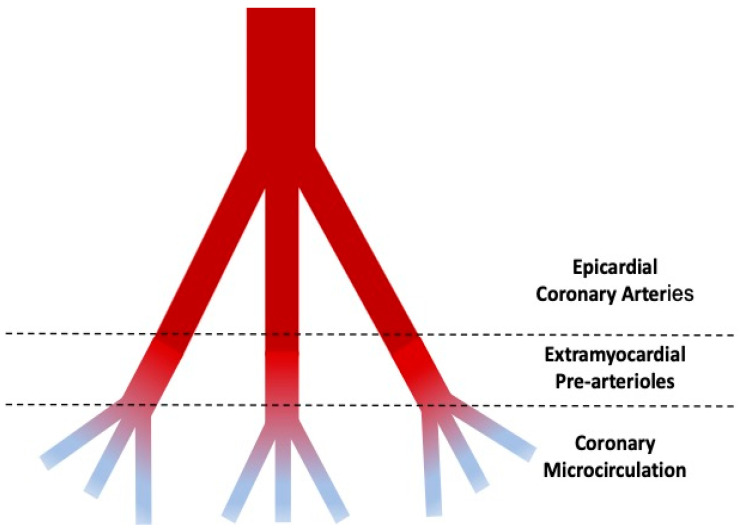
Schematic representation of the coronary circulation.

The coronary tree may be virtually divided into three compartments with decreasing size and different functions (Figure 1) [1].

The proximal compartment is represented by the epicardial coronary arteries, with a diameter ranging from 5 mm up to 0.5 mm, acting as adaptive vessels and offering just a little resistance to the flow. The intermediate compartment, including pre-arteriolar and arteriolar vessels with a diameter ranging from 0.5 to 0.1 mm, offers the most relevant part of resistance to the flow. The distal compartment is represented by capillaries, with a diameter < 0.1 mm and the function of providing oxygen to the myocardial wall. Intramyocardial pre-arterioles, arterioles, and capillaries constitute the coronary microcirculation.

## 3. Coronary Microcirculatory Dysfunction

CMD is emerging as a major cause of myocardial ischemia in symptomatic patients without obstructive coronary artery disease (INOCA). It is caused by several disorders affecting the structure and the function of the coronary microcirculation, leading to an inadequate blood supply, finally resulting in myocardial ischemia [2,3].

Functional mechanisms responsible for CMD may be related to the presence of an imbalance between vasodilative and constrictor forces acting on the coronary microcirculation. Impaired vasodilation can be caused by endothelium-dependent and/or endothelium-independent mechanisms [2,3,4,5]. While these latter are probably caused by an impairment of vascular smooth muscle cells (VSMCs) relaxation because of the increased release of vasoconstrictor agonists or the enhanced sensibility to normal vasoconstrictor stimuli, the former is related to endothelial dysfunction, particularly sensible to the cardiovascular risk factors, which may be responsible for a reduced production and/or enhanced degradation of NO and other endothelial-derived relaxation factors, resulting in blunted coronary flow increase or vasoconstriction. Vasomotor abnormalities can be unmasked only during invasive coronary angiography through the administration of intracoronary boluses of acetylcholine. CMD may also be a consequence of diffuse structural alterations such as luminal narrowing of the arterioles and capillaries, perivascular fibrosis, and capillary rarefaction [3]. In this case, CMD presents with impaired blood flow and increased coronary microvascular resistance during drug-mediated hyperemia. Of note, in some patients, structural remodeling and vasomotor disorders may concur and can even be present in the context of patients presenting with obstructive coronary artery disease. The main endotypes, with their pathophysiological mechanism, are described in Table 1.

## 4. The Functional Coronary Angiography

**Figure 2:**
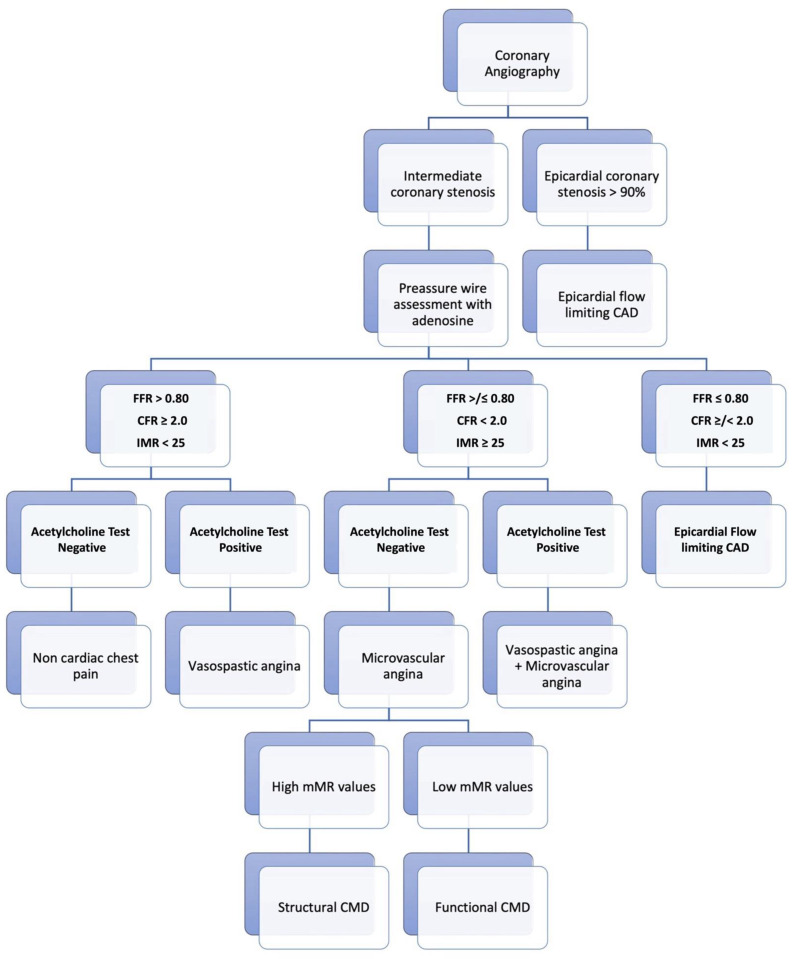
Functional coronary angiography flowchart.

While invasive coronary angiography relies on the visual estimation of the coronary tree, including sometimes the use of quantitative tools such as quantitative coronary angiography (QCA), functional coronary angiography (FCA) requires a direct invasive assessment of both the epicardial disease and the microcirculation, using different tools in combination with a pharmacological reactivity test, according to a “full physiology” approach (Figure 2) [6].

Blood flow through the coronary tree is continuously regulated with the aim of delivering an adequate blood and oxygen supply to the myocardium, either at rest or during exercise. The ability of the entire coronary bed to adequately respond to any increased myocardial oxygen demand, maintaining stable myocardial perfusion across a range of perfusion pressures, is called “autoregulation”, and the coronary flow reserve (CFR) describes the ratio by which coronary blood flow can be augmented by exercise, stress, or microcirculatory vasodilation [7]. Thereby, the measurement of the CFR allows for a comprehensive assessment of both epicardial and microvascular compartments. Of note, when a reduced CFR value is found, either the impaired microcirculation and/or the presence of myocardial bridging, as well as obstructive CAD, can be responsible [8]. For this reason, several indexes have been proposed to specifically assess the microcirculation, regardless of the presence of any disease of the epicardial compartment. This latter can also be assessed with specific tools able to hemodynamically evaluate the ischemic potential of coronary artery stenoses. The fractional flow reserve (FFR) is a well-validated diagnostic tool for invasively assessing the ischemic potential of the epicardial compartment, and it represents the gold standard for the functional assessment of intermediate coronary artery stenosis [9,10,11,12,13,14]. Functionally significant coronary artery stenosis can be identified with an FFR value ≤ 0.80. Of note, several non-hyperemic indexes requiring no administration of any hyperemic drugs, such as the instantaneous wave-free ratio (iFR) or the resting full-cycle ratio (RFR), have been recently introduced to simplify invasive functional evaluation and enhance clinical adoption [15,16,17,18]. Although with some limitations, a functionally significant epicardial coronary artery disease can be identified with a value < 0.90 [19,20,21].

In the presence of endothelial dysfunction, a dysregulation of the vasodilatory cascade in coronary resistance vessels may occur. Thus, when myocardial oxygen demands increase, the presence of endothelial dysfunction can be responsible for impaired vasodilation and even paradoxical vasoconstriction of upstream arteries and arterioles. A vasoreactivity test can be performed during FCA to highlight this phenomenon. The most established approach is represented by intracoronary infusion of acetylcholine, which influences coronary vascular tone via muscarinic receptors on endothelial and vascular smooth muscle cells [22,23,24,25]. Acetylcholine test is currently recommended by the ESC guidelines [26]. A standard approach involves sequential intracoronary administration of acetylcholine at concentrations increasing from 20 μg up to 200 μg [22]. However, some limitations associated with provocative tests should be acknowledged, as also clearly indicated by Lanza G.A. et al.: (1) Acetylcholine is normally used to assess endothelium-dependent dilatation; however, it has been shown that it also has a direct constrictor effect on SMCs, potentially contributing to abnormal coronary microvascular responses; (2) A global impairment of SMC relaxation in response to vasodilator substances can lead to a misdiagnosis of endothelium-dependent dilatation. Thereby, in some cases, it might be difficult to definitively attribute CMD to a specific mechanism [27].

A clinical diagnosis to rule in or rule out microvascular or epicardial spasm is made according to well-established criteria (Table 2 and Table 3) [23,28].

## 5. Assessment of Coronary Flow Reserve and Microcirculation

A comprehensive evaluation of the coronary physiology, namely the combination of epicardial and microcirculatory conduits, can be performed by measuring CFR, while several indexes can be measured to evaluate the microcirculation isolated from the upstream epicardial conduit. Although initially used for research purposes only, it is nowadays more often measured, particularly in patients with non-flow-limiting epicardial stenosis. Coronary flow reserve and microcirculation can be assessed using different tools and techniques: Doppler-based tools and thermodilution-based tools.

## 6. Doppler-Based Tools

Coronary flow velocity measurement by ultrasonic Doppler systems has provided crucial information to modern theories of coronary hemodynamics. A catheter-based Doppler system was previously introduced in the early 1970s by Benchimol, Hartley, and Cole [29,30,31]. Since then, many improvements have been made. The currently used device is a 0.018–0.014” flexible and steerable guidewire with a 12 MHz piezoelectric ultrasound transducer integrated onto its tip and a forward-directed ultrasound beam. It measures flow velocity in a sample volume of 2.25 mm in diameter, at 5.2 mm from its transducer. After the wire crosses the epicardial stenosis, it can assess flow velocity in small and distal branches of the coronary tree. This wire is proximally connected to a real-time spectrum analyzer, which records flow velocity during the whole cardiac cycle. Simultaneously, it can record additional parameters such as ECG track, aortic pressure (Pa), instantaneous spectral peak velocity, and the time average of spectral peak flow velocity (APV—cm/s). Velocity measurements are continuously displayed on the FloMap monitor in a gray-scale spectral pattern. Functional assessment is usually performed in the left anterior descending coronary artery (LAD) since it supplies a larger myocardial mass.

Douchette et al. and Labovitz et al. have previously validated the use of Doppler flow wire for the functional assessment of coronary hemodynamics in vivo by deriving quantitative coronary flow from the product of vessel cross-sectional area and mean velocity, assuming that a linear correlation between the average peak flow velocity (APV) and blood flow exists in straight vessels [32,33]. Doppler FloWire has also been used to measure CFR to assess the ischemic potential of intermediate coronary stenosis. CFR is calculated as the ratio between the hyperemic (hyp) APV, measured during drug-induced maximal hyperemia, and resting (rest) coronary flow velocity (APV_hyp_/APV_rest_). Maximal hyperemia is achieved by i.v. adenosine administration (140 µg/Kg/min). CFR values ≤ 2.5 are considered abnormal, with a negative prognostic value, predicting major cardiovascular events at long-term follow-up [34,35].

Doppler flow wire has also been used to directly assess the hyperemic microvascular resistance (HMR—mmHg·cm^−1^·s), a microvascular-specific index. This is defined as the ratio between the distal coronary pressure (Pd) and the APV during maximal hyperemia [36]. Of note, this formula needs to be corrected for collateral flow in case of the presence of obstructive CAD [37].

However, the cutoff value of HMR for prediction of CMD is still a matter of debate. A study including symptomatic patients with non-obstructive CAD proposed an HMR value ≥ 2.5 to identify patients with CMD [38].

Minimal microvascular resistance (mMR—mmHg·cm^−1^·s) is another Doppler-based invasive method for the assessment of coronary microvascular resistance, and it is calculated by assessing both Pd and APV during the diastolic wave-free (WF) period and during maximal hyperemia, using the following formula:

$$\frac{Pd(WFperiod)}{APV(WFperiod)}during\;maximal\;hyperemia$$

It has been developed to overcome the limitations of HMR in the presence of obstructive CAD. In this setting, HMR can overestimate microvascular resistance. De Waard et al. showed that mMR provides a reliable evaluation of the coronary microcirculation status, regardless of the presence of epicardial stenosis [39]. For this reason, mMR can be useful in assessing microvascular disease in patients presenting with either obstructive or non-obstructive coronary artery disease. Moreover, mMR might be used with CFR to discriminate structural from functional CMD. Low CFR and increased mMR values indicate structural CMD. On the contrary, low CFR and reduced mMR values allow the identification of functional CMD [40].

## 7. Advantages and Limitations of Doppler-Based Tools

Doppler-based tools provide numerous advantageous features: low operator dependency, low risk for the patient, and high diagnostic accuracy. Doppler devices are also relatively widely available in catheterization laboratories.

Furthermore, the employment of Doppler guidewires results in a minor degree of obstruction in small vessels, causing only a negligible disturbance of Doppler flow velocity profiles. Additionally, when performing percutaneous coronary intervention (PCI), the Doppler wire may serve as both a diagnostic and intervention tool [32].

Beyond their many advantages, Doppler-based tools require overcoming some limitations. In general terms, both the quality of the Doppler signal and the value of peak velocity are particularly dependent on the correct positioning of the wire, which may limit the use of a Doppler device in large vessels or in segments with variable luminal caliber [32]. Values obtained with this technique may also differ because of loading conditions (i.e., heart rate, blood pressure, left ventricular contractility) [38].

When compared to thermodilution-based CFR, Doppler-derived CFR has the advantage of having a greater agreement with oxygen-15-labeled water PET-derived CFR, which is considered to be the gold standard for the quantification of CFR [41]. The use of HMR is limited by the lack of a determined cutoff value. As previously stated, it is still unclear whether the HMR reference value should be ≥ 1.9 or ≥2.5 [38]. Both CFR and HMR require adenosine-induced hyperemia. In contrast, i.v.-infused and i.c.-injected adenosine may cause several side effects ranging from negligible to significant. The advantages and limitations of Doppler-based tools are summarized in Table 4.

## 8. Clinical Evidence in Non-Acute Scenarios

Myocardial ischemia is due to both epicardial stenoses and microvascular dysfunction, which is emerging as a new pathogenic mechanism responsible for non-obstructive coronary artery disease and several other conditions, including non-ischemic cardiomyopathies, takotsubo syndrome, and heart failure, particularly with preserved or mildly reduced ejection fraction. FloWire can be used to measure both CFR and HMR in order to assess microvascular function when the epicardial compartment is free from obstructive disease [42]. Increased microvascular resistance is considered a marker of coronary microvascular dysfunction and is associated with worse clinical outcomes.

Feenstra et al. enrolled a cohort of 41 patients presenting with angina, non-obstructive coronary artery disease (<50% luminal obstruction on angiography), FFR > 0.80, and CFR > 2.5 undergoing vasomotor function test. They aimed to establish a reference range for Doppler flow velocity-derived index HMR, and they documented that values equal to or higher than 2.5 mmHg/cm/s can identify increased microvascular resistance and, therefore, microvascular dysfunction, regardless of patients’ clinical features [43].

Both Doppler wire-derived CFR (CFR_doppler_) and HMR have been respectively compared with thermodilution-derived CFR (CFR_thermo_) and IMR. Demir et al. enrolled 250 vessels (149 patients) in their study. A modest correlation was found between CFR_thermo_ and CFR_doppler_ (r^2^ = 0.36; *p* < 0.0001). In particular, CFR_thermo_ overestimates CFR_doppler_ (mean 2.59 ± 1.46 vs. 2.05 ± 0.89; *p* < 0.0001). In addition, a CFR_thermo_ threshold of 2.0 had poor sensitivity for identifying vessels with diminished CFR, but using the same Doppler threshold (<2.5), diagnostic accuracy increased [44]. Williams et al. compared HMR vs. IMR and both with independent measurements of CMD. A total of 54 patients (61  ±  10 years) who underwent cardiac catheterization for stable coronary artery disease (*n*  =  10) or acute myocardial infarction (*n*  =  44) were enrolled. They found that an HMR of ≥2.5 mmHg/cm/sec and an IMR from 21.5 to 24 were optimal thresholds to define CMD compared to the average values of CFR_thermo_ and CFR_doppler_ and cardiac magnetic resonance (CMR) [38]. Moreover, HMR showed better diagnostic accuracy compared to IMR, CFR (both CFR_doppler_ and CFR_thermo_), and CMR in detecting CMD.

## 9. Clinical Evidence in Acute Scenarios

In the setting of acute coronary syndromes (ACS), up to 50% of patients presenting with ST segment elevation myocardial infarction (STEMI) develop microvascular injury (MVI) despite angiographically successful primary percutaneous coronary intervention (PCI). Different pathogenic mechanisms after coronary revascularization play a role, such as distal embolization of the thrombus, endothelial dysfunction, reperfusion injury, and intramyocardial hemorrhage.

In this setting, Doppler FloWire can be used to detect patients who still have impaired flow at the myocardial microcirculatory and after percutaneous revascularization. Microvascular obstruction (MVO) occurs with reduced coronary flow velocity reserve, systolic flow velocity reversal, and a short diastolic deceleration time. A correct diagnosis of MVO could lead these patients to benefit from adjunctive therapy after primary PCI.

Teunissen et al. demonstrated that in patients presenting with STEMI, HMR values > 2.5 mmHg/cm/s correlate with MVI (as assessed by CMR) and with decreased PET myocardial blood flow after primary PCI. Microvascular resistance indexes can be immediately performed in a catheterization laboratory, allowing for an early risk stratification and management of microvascular damage, whereas CMR and PET are usually only performed after the index procedure. The role of HMR on long-term clinical outcomes in patients with STEMI undergoing PCI was investigated for the first time by Jin et al. In this study, HMR was found to be a strong predictor of long-term major adverse cardiovascular events [45].

An additional study by de Waard et al. demonstrated that HMR is more reliable in identifying patients with ACS and high risk of adverse clinical events immediately after primary percutaneous revascularization, compared to CFR_doppler_. Indeed, HMR > 3.0 mmHg/cm/s had a predictive value for the composite end point (HR 7.0; 95% CI 1.5 to 33.7) as well as both individual components (death and hospitalization for heart failure), whereas CFR_doppler_ only had it for the composite endpoint (HR 7.0; 95% CI 1.5 to 33.7) [46].

Finally, in a recent metanalysis by Canu et al., it was confirmed that severe CMD, defined as IMR > 40 mmHg or HMR > 3 mmHg/cm/s, was associated with increased risk of long-term adverse cardiovascular events in patients with STEMI, suggesting that both IMR and HMR can have an important prognostic role and guide therapeutic approach [47].

## 10. Thermodilution-Based Tools

Indicator-dilution theory and the “Stewart-Hamilton” equation represent the basis for measuring the flow in a human district [48,49]. The “indicator” can be represented by a traceable substance with physical properties (i.e., temperature, pH) that can be measured while diluted with blood. In addition, a specific device is also required to measure the “passage” of the indicator during time. This theory is normally applied for the assessment of the cardiac output, which is measured with a Swan–Ganz catheter placed in the pulmonary artery, which can measure the variations of blood temperature during the time and allows injecting saline at room temperature as a “colder than blood” indicator.

**Figure 3:**
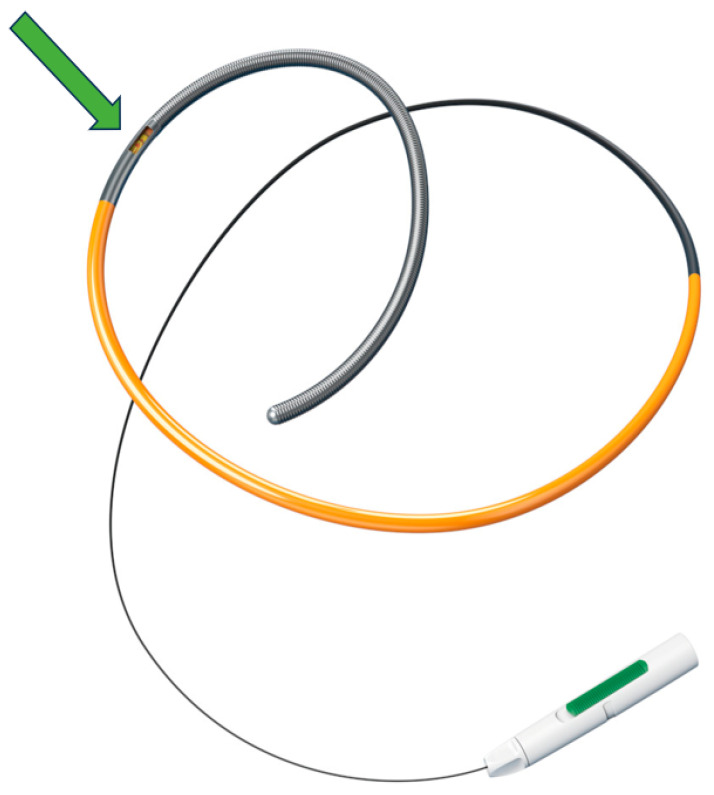
Abbott Cardiovascular Pressure Wire X. Green arrow: pressure and temperature sensor.

In the coronary system, the colder-than-blood indicator can be administered either as a bolus injection or continuous infusion. In both cases, a specific pressure–temperature coronary wire must be used to detect both the distal coronary pressure and the temperature difference while the indicator is diluted (Figure 3).

## 11. Thermodilution by Bolus Injection

**Figure 4:**
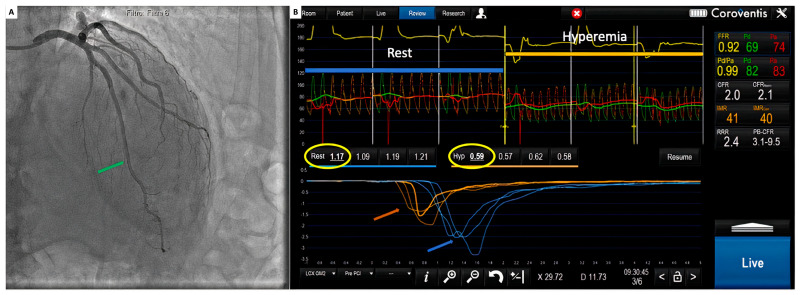
(**A**) Coronary angiogram. Green arrow: pressure wire sensors; (**B**) coroventis interface during invasive functional assessment. Red and green pressure tracings represent, respectively, aortic (Pa) and distal coronary pressure (Pd). Blue arrow: intracoronary temperature variation of 3 consecutive boluses of saline at rest. Orange arrow: intracoronary temperature variation of 3 consecutive boluses of saline during maximal hyperemia. Yellow circles: average mean transit time values at rest (Rest) and during hyperemia (Hyp).

With a 6 Fr guiding catheter positioned in the coronary ostium, a 0.014″ floppy pressure–temperature coronary guidewire is advanced through the coronary artery, after equalization, and located as distal as possible into the target vessel. Thermodilution curves can be obtained with a short manual injection of 3 to 5 mL of saline at room temperature, and a time-based temperature change can be recorded in the distal part of the artery (Figure 4).

Because of the low reproducibility, measurements must be performed three times, taking care not to move the wire during the injections, during two different periods: at baseline and during maximal hyperemia [50].

When bolus thermodilution is used to assess coronary flow, the exact volume of the indicator is not known. In fact, a portion of the injected saline can go lost in the aorta during bolus injection. In this case, the coronary flow (Q−mL/s) can be approximately calculated by dividing the vascular volume (V−in mL) by the mean transit time of the indicator (Tmn; in seconds):

$$Q=\frac{V}{Tmn}$$

However, vascular volume (V) is also unknown, but it can be considered constant during consecutive measurements and under hyperemic conditions. In this case, Tmn can be considered inversely proportional to Q:

$$Q=\frac{1}{Tmn}$$

The mean transit time (Tmn) can be finally calculated from thermodilution curves and, as the index of flow, when measured both at rest and during maximal hyperemia, it can be used to calculate coronary flow reserve (CFR):

$$\mathrm{CFR}=\frac{Q\;hyp}{Q\;rest}=\frac{Tmn\;rest}{Tmn\;hyp}$$

Experimental models have previously found a significant agreement between bolus thermodilution-based CFR and Doppler-based CFR [50,51]. Furthermore, CFR_thermo_ is also technically easier to perform than CFR_doppler_ [52].

Commonly, a value lower than 2 is considered abnormal, providing also a prognostic role. However, more recently, some authors suggested using a cutoff of 2.5, which is similar to the one suggested for the CFR_Doppler_ in order to improve the diagnostic accuracy [35].

Bolus thermodilution can also be used to directly evaluate the microvascular compartment through the assessment of the index of microcirculatory resistance (IMR), which is defined as the ratio of the distal coronary pressure and the inverse of Tmn during maximal hyperemia:

$$\mathrm{IMR}=\frac{Pd(hyp)}{\frac{1}{Tmn(hyp)}}=Pd\left(hyp\right)\times Tmn(hyp)$$

This index was introduced to specifically evaluate the microvascular resistance as a single compartment independent of the epicardial section, thereby specific for coronary microcirculation regardless of the presence of epicardial disease. In an animal model, Fearon and colleagues showed a suitable correlation between IMR and true microcirculatory resistance (TMR), defined as distal coronary pressure divided by absolute coronary flow during maximal hyperemia. In addition, IMR was able to distinguish between normal and abnormal microcirculatory function, this latter simulated using embolized microspheres [53].

## 12. Catheterization Protocol

With a 6 Fr guiding catheter placed in the coronary ostium, a 0.014″ floppy pressure–temperature guidewire is connected to the pressure analyzer and calibrated outside the body, then it is advanced to the tip of the guiding catheter to check the equality of pressure signals. The guide catheter is flushed with saline, clearing all contrast, and after the equalization, the temperature signal is calibrated so the temperature at the coronary ostium is taken as a reference for measurements. Next, the wire is advanced into the coronary artery and located as distally as possible in the target vessel. At this point, three boluses of 3 to 5 mL of saline at room temperature are injected both at baseline and during maximal hyperemia, normally obtained with intravenous infusion of adenosine. In this way, two couples of triple thermodilution curves can be produced, representing each of them the mean transit time values obtained three times at baseline and during hyperemia. At the same time, both Pa and Pd are recorded at rest and during maximal hyperemia. In this way, FFR, CFR, and IMR are all measured at the same time, allowing the operator to contemporarily assess the epicardial compartment and the coronary microcirculation [51,53,54].

## 13. Advantages and Limitations of Bolus Thermodilution

Thermodilution-derived CFR tends to incorporate the same limitations of Doppler-derived CFR: containing resting flow in its mathematical formula, any hemodynamic change, such as different loading conditions or epicardial coronary artery disease, may result in a lack of reproducibility and a greater degree of variability [54].

Both CFR and IMR require adenosine-induced hyperemia, with the potential side effects already mentioned. Unlike CFR, IMR values are not altered by different hemodynamic states.

Although being performed through manual indicator injections, IMR values measurement remain consistent, with remarkable intraoperator and interoperator reproducibility. Furthermore, Tmn persists to be stable regardless of the indicator temperature or volume injected [41].

Unless subocclusive, epicardial coronary stenosis results in a negligible alteration of IMR values. In this case, due to an underestimation of coronary flow, IMR measurements obtained may be falsely higher. To overcome this limitation, it is necessary to take into account collateral flow by assessing coronary wedge pressure (Pw) and integrating the result in a new formula, as follows:

$$\mathrm{IMR}=\mathrm{Pa}\;\times \;\mathrm{TmnHyp}\;\times \;\frac{Pd-Pw}{Pa-Pw}$$

Lastly, Tmn can vary according to the distance of the thermistor from the ostium of the coronary artery; hence, movement of the sensor greater than 2 cm might disrupt Tmn evaluation [54]. The advantages and limitations of bolus thermodilution are summarized in Table 4.

## 14. Clinical Evidence in Non-Acute Scenarios

Patients experiencing chest pain often show non-obstructive epicardial coronary artery disease during invasive coronary angiography (ICA). These patients may present with exertional angina; noninvasive tests positive for the evidence of inducible ischemia; or either no epicardial stenosis, as previously stated, or mild to moderate stenosis (ranging from 40 to 60%) unfolded by ICA or computed tomography angiography (CTA), that are accounted to be functionally not significant [26].

In this subset of patients, CMD is strikingly prevalent [41]. This condition is mainly linked to women [55], associated with poor clinical outcomes, and deserves targeted therapy. In 2003, Fearon et al. established the index of microvascular resistance as the first index to assess specifically the microcirculation [53]. In apparently healthy subjects, several examinations suggested that normal IMR reference values are generally lower than 25 [54,55].

In 2015, Lee et al. [56] assessed IMR in 1096 patients, demonstrating different cutoff values for each major coronary artery: 22.0 for the left anterior descending (LAD) coronary artery, 24.0 in the circumflex coronary artery, and 28.0 in the right coronary artery (RCA). The greater value detected in the RCA might be related either to the longer length of the vessel (and consequently to a longer mean transit time) or the smaller amount of subtended myocardial mass [57].

Medical therapy, stratified according to an invasive physiologic assessment consisting of IMR and CFR, can improve typical chest pain relief after 6 months in patients with angina and non-obstructive coronary artery disease compared with standard care [25].

In addition, IMR can serve as a predictor of myocardial injury after percutaneous coronary intervention. In patients presenting with chronic coronary syndrome, higher IMR values (detected pre- and post-PCI) are strong predictors of the occurrence of type 4a myocardial infarction [58].

## 15. Clinical Evidence in Acute Scenarios

When employed in acute scenarios, such as STEMI, and performed after a successful primary PCI, IMR can predict the presence of MVO, the extension of the infarct size, and which patients are at risk of major cardiac complications within 30 days [41]. MVO is the leading mechanism of no reflow, causing the persistence of deteriorated myocardial perfusion in patients presenting with STEMI who have already undergone a successful epicardial recanalization of the culprit coronary artery [59].

CMR is often regarded as the gold standard for assessing MVO but is not always easily available and performed after the acute phase. It may provide the proper information but not right after the coronary revascularization, i.e., when it is most needed [60]. On the other hand, IMR can be readily measured in the catheterization laboratory and could allow for earlier administration of adjunctive therapy [54].

Ahn et al. investigated the predictive accuracy of invasive microvascular indexes, such as IMR and CFR_thermo_, for identifying MVO versus CMR in patients presenting with STEMI undergoing primary PCI. IMR and CFR_thermo_ were assessed in 40 STEMI patients soon after successful primary PCI and compared with CMR assessed within 7 days. The investigators found that high IMR values and low CFR_thermo_ values were associated with MVO. In contrast, low IMR values and high CFR_thermo_ values suggested preserved microvascular function. The combination of both indexes performed better than the use of one of them [61].

## 16. Thermodilution by Continuous Infusion

The assessment of microvascular resistance based on actual coronary blood flow measurements has recently become possible by continuous thermodilution with intracoronary saline infusion, using a pressure–temperature guidewire and an infusion microcatheter. The method was validated in vitro, in animals, and in humans [62,63].

Recent improvements in hardware and software enabled the technique to be introduced into routine clinical practice [64,65]. The basic principle of continuous thermodilution has been recently validated and proved to be operator independent; furthermore, it is not necessary to use pharmacological agents to induce maximal hyperemia [66]. In fact, a recent study indicates that saline-induced hyperemia is mediated by hemolysis, either by rupture or deformation of red blood cells (RBCs), and suggests that vasodilatory compounds, including ATP and NO, are released locally from RBCs and are ultimately responsible for maximal steady-state vasodilation [67].

Absolute blood flow can be calculated with the following equation:

$$\begin{array}{c} Q=1.08\frac{T_{i}}{T}Q_{i} \end{array}$$

where Q represents the hyperemic coronary blood flow (mL/min); $Q_{i}$ represents the indicator flow (saline at room temperature—mL/min); T represents the difference in temperature in °C between body temperature (calibrated to 0) and the mixture of blood and saline, and it is measured by the pressure wire in the distal part of the coronary artery; $T_{i}$ represents the difference in temperature in °C between body temperature and saline only, and it measured when the pressure wire is pulled back in the infusion catheter. The constant 1.08 accounts for the densities and specific heat of blood and saline [41]. Pd is also recorded simultaneously, so microvascular resistance (R) can be calculated by dividing distal pressure and flow according to the following equation:

$$\begin{array}{c} R=\frac{Pd}{Q} \end{array}$$

**Figure 5:**
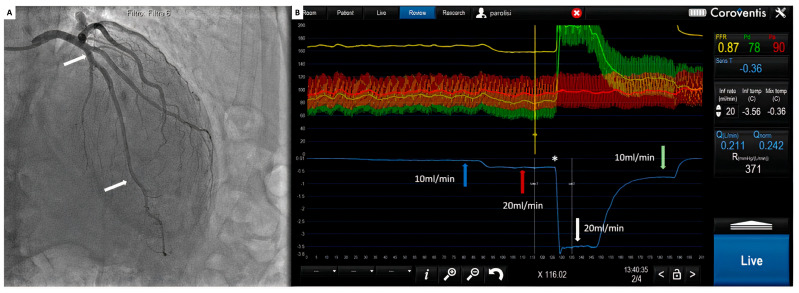
(**A**) Coronary angiogram. White arrows: tip of the infusion microcatheter at the proximal part of the LAD and pressure–temperature wire at the distal part; (**B**) coroventis during continuous thermodilution. Blue arrow: temperature during infusion at 10 mL/min; red arrow temperature during infusion at 20 mL/min; *: pressure wire pull back; white arrow: temperature of the saline in the infusion microcatheter; green arrow: temperature during 10 mL/min.

All signals are instantaneously recorded by a dedicated software (Coroflow^®^ v3.0 Coroventis, Uppsala, Sweden), which can measure all pressure parameters, fractional flow reserve as well as absolute blood flow in mL/min and microvascular resistance in mmHg/L/min or wood units (WU) (Figure 5).

Moreover, continuous thermodilution with infusion of saline at the low rate of 10/mL allows for the assessment of CFR (CFR_abs_) and microvascular resistance reserve (MRR). CFR_abs_ is calculated by dividing Q during maximal hyperemia (Q_h_) and Q at rest (Q_r_):

$$CFRabs=\frac{Qh}{Qr}$$

MRR is calculated according to the formula shown by De Bruyne et al., as follows:

$$MRR=\frac{CFR}{FFR}\times \frac{Pa\;rest}{Pa\;hyp}$$

and it can be assessed both with bolus and continuous thermodilution methods. However, hyperemic Pa (Pa_hyp_) can be lower when assessed by bolus thermodilution because of adenosine administration, as compared with continuous infusion. Thereby, an overestimation of the MRR values might occur with bolus thermodilution [68].

Interestingly, Jansen et al. demonstrated that, unlike bolus thermodilution-derived CFR and MRR, absolute flow measurements correlate well with angina severity and overall quality of life [69].

## 17. Catheterization Protocol

Cardiac catheterization and FFR measurement can be performed as usual by either radial or femoral access. Guiding catheters and a pressure/temperature wire (Pressure wire X™, Abbott, Saint Paul, MN, USA) can be manipulated as a regular practice. After the intracoronary administration of 200 micrograms of nitroglycerin and proper equalization of pressures, the pressure wire can be advanced into the coronary artery. A dedicated monorail infusion catheter (Rayflow™, Hexacath, Paris, France) can be advanced over the pressure wire and positioned with its tip at the proximal part of the coronary artery [70]. This infusion catheter (2.5 French) consists of a 25 cm long rapid exchange inner monorail lumen for the 0.014″ wire, and it is equipped with four infusion holes guaranteeing rapid and complete mixing of saline with blood in the coronary artery. In addition, the infusion catheter has two inner side holes, which allow the recording of the temperature of saline at the site where it enters the coronary artery. Before saline infusion starts, the temperature is calibrated, and body temperature is set to “zero” (reference temperature); thereafter, all changes in temperature are related to this reference temperature (Figure 5). During the measurement, the pressure–temperature sensor of the pressure wire is positioned in the distal part of the coronary artery. Then, saline infusion is started at the rate of 10 to 20 mL/min, inducing maximal hyperemia within 10–20 s. After obtaining a steady state, the temperature of the completely mixed blood and saline (T) is measured in the distal coronary artery; the pressure/temperature wire is pulled back into the tip of the infusion catheter to measure the saline temperature (Ti) [71].

## 18. Advantages and Limitations of Continuous Thermodilution

The continuous thermodilution method showed several advantages over other coronary assessment techniques. Primarily, it has been demonstrated to be safe and reproducible [72]. Xaplanteris et al. observed no significant periprocedural adverse events in 203 absolute coronary blood flow (Q) and resistance (R) measurements in 135 patients undergoing clinically indicated coronary angiography [65]. A small percentage (8.1%) of patients experienced transient bradycardia and concomitant atrioventricular block, which were reversed immediately upon interruption of the infusion.

Keulards et al. observed one catheter-induced coronary dissection requiring percutaneous coronary intervention in 467 absolute Q and R measurements from 100 consecutive patients undergoing clinically indicated invasive fractional flow reserve measurements [73]. They observed only 2.6% cases of bradycardia and atrioventricular block and two patients experiencing transient chest discomfort during infusion without electrocardiographic abnormalities. Xaplanteris et al. also assessed reproducibility in their cohort [65]. Duplicate measurements were performed in 80 patients, which had a strong correlation both for Q (ρ = 0.841; *p* < 0.001; ICC: 0.89; *p* < 0.001) and R (ρ = 0.780; *p* < 0.001; ICC: 0.89; *p* < 0.001). This methodology is operator independent, and it does not require adenosine infusion because saline infusion directly induces maximal hyperemia. Of note, differently from adenosine-induced hyperemia, the one induced by saline infusion does not cause chest discomfort in most patients [73]. Another strength of continuous thermodilution is that it has been validated against the current gold standard for flow assessment, PET-TC [74].

However, there are limitations associated with this microvascular assessment method, the most important of which is that there is significant interpatient variability in Q and R during hyperemia owing to the difference in the vascular territory supplied and, hence, correction for myocardial mass using computed tomography may be needed, and no well-accepted normal values are available [74,75]. Moreover, the clinical impact of the hemolysis process is yet to be fully assessed.

In a recent sub-study by Jansen et al., 73 patients underwent coronary functional tests (acetylcholine test, CFR_thermo_, IMR, Q, and R) at baseline and after 6 weeks. Continuous thermodilution-derived Q and R show better reproducibility than CFR and IMR [76]. The advantages and limitations of this technique are summarized in Table 4.

## 19. Clinical Evidence in Non-Acute Scenarios

Konst et al. have explored the relation between Q and R and CFR and IMR and the relation with self-reported angina. In 84 enrolled patients, they found that the absolute R value was higher in patients with CMD, defined as abnormal CFR or IMR, than in the control group (495 WU vs. 374 WU); absolute Q was not different between these groups (191 mL/min vs. 208 mL/min) [77].

Furthermore, they observed that low Q (<198 mL/min) and high R (>416 WU) were associated with the severity of angina. Therefore, continuous thermodilution-derived measurements correlated with microvascular dysfunction and angina but were not associated with endothelium-dependent coronary spasm during acetylcholine testing.

Similarly, Rivero et al. demonstrated that Rmicro derived from continuous intracoronary thermodilution represents an accurate technique to assess the microvascular function and correlates well with IMR. An Rmicro value above 500 WU measured in the proximal LAD accurately predicts the presence of CMD. However, the reference values for absolute Q and R need to be validated in larger studies and correlated with clinical outcomes, potentially representing a future guide in patient-tailored therapy and an essential tool for the INOCA diagnostic workup [71].

Scarsini et al. used MRR to assess CMD in patients with severe aortic stenosis undergoing transcatheter aortic valve implantation (TAVI). They assessed coronary microcirculation at baseline and promptly after the TAVI procedure and found that CMD correlated with a low-flow phenotype and with extra-valvular cardiac damage (EVCD) related to severe aortic stenosis [78].

## 20. Clinical Evidence in Acute Scenarios

Recently, Wijnbergen et al. demonstrated that continuous thermodilution, using an intracoronary infusion of saline, permits safe and feasible measurement of absolute coronary blood flow and microvascular resistance during primary PCI in STEMI patients. This technique may allow better exploration and understanding of microvascular dysfunction in the early phase of STEMI. Absolute blood flow per gram of tissue of myocardium at risk correlates well with IMR. During the first couple of days, absolute flow increases and resistance decreases in a significant number of patients, indicating recovery of microvascular dysfunction over time. Larger studies are required to evaluate the clinical use of continuous thermodilution-derived indexes in acute settings [79].

## 21. Conclusions

With the advancement of technology, numerous tools are available to assess the microcirculation. Although microvascular angina benefits from its own definite therapy, the techniques for diagnosing it are often overlooked and underemployed. Contrarily, the use of these methods should be implemented in the diagnostic workup of INOCA patients to promptly establish an appropriate therapeutic regimen. At last, larger randomized clinical trials are necessary to appraise the clinical use of the latest and most advanced continuous thermodilution techniques.

## Figures and Tables

**Table 1:** INOCA endotypes with diagnostic criteria.

Endotypes	Mechanism	FFR	CFR	IMR	Vasoreactivity	mMR
**Microvascular Angina**	CMD	±	<2	≥25	<90% diameter reduction	
**Structural**						High values
**Functional**					Microvascular spasm	Low values
**Vasospastic Angina**	Epicardial spasm	±	≥2	<25	≥90% diameter reduction	N/A
**Microvascular And Vasospastic Angina**	Both CMD and epicardial spasm	±	<2	≥25	≥90% diameter reduction	

CFR: coronary flow reserve; CMD: coronary microvascular dysfunction; FFR: fractional flow reserve; IMR: index of microcirculatory resistance; mMR: minimal microvascular resistance; N/A: not applicable.

**Table 2:** The Coronary Vasomotion Disorders International Study Group (COVADIS) criteria for microvascular angina. Definitive MVA is diagnosed if all four criteria are fulfilled. Any evidence of microvascular dysfunction can be present to support the definitive diagnosis. Suspected MVA is diagnosed if three out of four criteria are present, including symptoms with evidence of non-obstructive coronary artery disease.

Symptoms of Myocardial Ischemia	Absence of Obstructive CAD	Objective Evidence of Ischemia	Evidence of Microvascular Dysfunction
Effort/rest angina	Coronary CTA	Ischemic ECG changes during spontaneous episode of chest pain	Impaired CFR
Angina equivalents (dyspnea)	Invasive coronary angiography	Stress-induced chest pain and/or ECG changes and/or abnormal myocardial perfusion wall motion abnormalities	Microvascular spasm (defined as reproduction of symptoms and ischemic ECG changes, but no epicardial spasm during acetylcholine testing)
			IMR > 25
			Coronary slow-flow phenomenon (TIMI frame count > 25)

**Table 3:** The Coronary Vasomotion Disorders International Study Group (COVADIS) criteria for vasospastic angina. Definitive VSA is diagnosed if, during nitrate-responsive angina, either ECG changes or coronary spasm criteria are fulfilled. Otherwise, if ECG changes and coronary vasospasm are equivocal, “Suspected vasospasm” is diagnosed.

Nitrate-Responsive Angina	Transient Ischemic ECG Changes	Coronary Artery Spasm
Rest angina (especially night/early morning)	ST segment elevation ≥ 0.1 mV	Transient total or subtotal coronary artery occlusion (>90%) with angina and ischemic ECG changes either spontaneously or in response to a provocative stimulus (acetylcholine, ergotamine)
Diurnal variation in exercise tolerance	ST segment depression ≥ 0.1 mV	
Hyperventilation precipitates the episode	New negative U waves	
Calcium channel blockers suppress episode		

**Table 4:** Advantages and limitations of invasive tools for CMD assessment (all three packages of invasive tools are described in the table).

	Doppler-Based Tools	Thermodilution by Bolus Injection	Thermodilution by Continuous Infusion
High diagnostic accuracy	+	+	++
Low operator dependency	+	+	++
Low risk for the patient	+	+	+
Available in the cath-lab	−/+	+	−/+
Requires hyperemic stimulus	+	+	−
Cutoff values	HMR (mmHg·cm^−1^·s) (1.9 or 2.5) CFR (2.5) mMR (mmHg·cm^−1^·s) (2.1) *	IMR (25) CFR (2.0)	Q (200 mL/min) * R (500 UW) * CFR (2.0–2.5) *
Loading condition dependent	HMR (−) CFR (+)	IMR (−) CFR (+)	Unknown
Advantages		Tmn remains stable regardless of the indicator’s temperature or volume injected.	Does not require injection of any hyperemic stimulus Higher reproducibility Provides indexes with unit of measure (mL/min and WU)
Disadvantages		Collateral flow must be assessed when significant epicardial stenosis is present in order not to overestimate IMR.	Requires specialized equipment and microcatheter Outcomes data are lacking

*: Under investigation.
